# Supplementary material for: Current forest carbon fixation fuels stream CO2 emissions
Source: Nat Commun. 2019 Apr 23;10:1876. doi: 10.1038/s41467-019-09922-3 (PMC6478898; doi:10.1038/s41467-019-09922-3)
Supplement: Supplementary file 1 — Supplementary Information [file 41467_2019_9922_MOESM1_ESM.pdf]

Supplementary Information

Current Forest Carbon Fixation Fuels Stream CO_2 Emissions

Campeau et al.

Supplementary Table 1: Summary of radiocarbon CO_2 samples collected with the passive molecular sieves in the stream over a complete year. The date of installation and retrieving of the passive sieves are noted, along with the volume of CO_2 recovered during the sampling period, and carbon isotope values. [#]The $\delta^{13}C$ values outside of the brackets are the measured values for CO_2 recovered from molecular sieve traps and were used to normalise the radiocarbon results. The $\delta^{13}C$ values within brackets have been corrected for isotopic fractionation that occurs during passive sampling and are representative of the CO_2 in the environment being sampled (Garnett et al. 2012).

Starting Date	Ending Date	Sampling Period (days)	Stream Sampling Location	Publication Code (SUERC-)	^{14}C-CO_2 enrichment (%modern ± 1σ)	Radiocarbon age (year BP ± 1σ)	$\delta^{13}C$-CO_2 (±0.1‰)[#]	Volume Retreived (ml)
2015-05-05	2015-06-08	36	Upstream	61846	103.98 ±0.45	Modern	-25.7 (-21.7)	3.7
2015-06-08	2015-08-04	58	Upstream	62409	103.72 ±0.48	Modern	-27.1 (-23.1)	12.7
2015-07-08	2015-08-06	30	Upstream	62403	103.69 ±0.48	Modern	-25.3 (-21.3)	9.5
2015-08-08	2015-09-02	26	Upstream	64035	104.40 ±0.64	Modern	-27.1 (-23.1)	12.2
2015-09-02	2015-10-12	40	Upstream	64701	104.87 ±0.46	Modern	-26.9 (-22.9)	14.0
2015-10-12	2015-11-24	44	Downstream	NA	NA	NA	NA	NA
2015-11-24	2016-01-19	57	Downstream	68170	102.50 ±0.47	Modern	-21.4 (-17.4)	12.0
2016-01-19	2016-03-30	72	Downstream	67423	105.19 ±0.48	Modern	-26.9 (-22.9)	22.4
2016-03-30	2016-05-11	43	Downstream	67980	104.22 ±0.48	Modern	-26.4 (-22.4)	10.9
2016-05-11	2016-06-14	36	Upstream	NA	NA	NA	NA	NA

* NA indicates samples that were lost due to cracks in the molecular sieve cartridge

Supplementary Table 2: Summary of manual spot measurements of radiocarbon composition of DOC and CO_2 collected with the super headspace method in the soil and stream water over the study year, including sample location, date of collection, carbon isotope values and corresponding C concentration.

Sample Location	Collection Date	Publication Code (SUERC)	$^{14}C-CO_2$ enrichment (%modern ± 1σ)	Radiocarbon age (year BP ± 1σ)	$\delta^{13}C-CO_2$ (±0.1‰)	CO_2 (mg C L^{-1})	Publication Code (SUERC)	^{14}C-DOC enrichment (%modern ± 1σ)	Radiocarbon age (year BP ± 1σ)	$\delta^{13}C$-DOC (±0.1‰)	DOC (mg C L^{-1})
Stream	2015-05-05	61387	105.29±0.46	Modern	-20.4	3.7	62147	112.21±0.49	Modern	-28.9	13.1
Riparian Shallow	2015-05-05	61383	106.60±0.47	Modern	-25.1	17.2	62144	116.11±0.53	Modern	-28.1	25.8
Riparian Deep	2015-05-05	61382	103.13±0.45	Modern	-25.0	8.2	62143	105.46±0.48	Modern	-27.9	18.2
Upslope Shallow	2015-05-05	61386	106.37±0.68	Modern	-23.7	3.8	62146	114.73±0.53	Modern	-28.0	6.0
Upslope Deep	2015-05-05	61384	105.48±0.49	Modern	-23.7	3.0	62145	116.83±0.53	Modern	-28.3	3.7
Stream	2015-08-08	64025	102.58±0.47	Modern	-22.8	4.5	63892	110.23±0.50	Modern	-28.3	22.1
Riparian Deep	2015-08-08	64023	99.05±0.45	77 +/- 37	-23.1	10.4	63890	100.80±0.46	Modern	-27.8	27.4
Upslope Deep	2015-08-08	64024	104.28±0.48	Modern	-25.0	16.1	63891	49.69±0.23	5617 +/- 37	-33.7	2.8
Stream	2015-10-12	64820	103.29±0.47	Modern	-23.5	10.9	65882	108.16±0.47	Modern	-28.0	16.1
Riparian Deep	2015-10-12	64818	101.08±0.44	Modern	-21.9	10.6	65880	102.63±0.47	Modern	-27.9	51.3
Upslope Deep	2015-10-12	64819	101.80±0.47	Modern	-24.2	4.0	65881	67.76±0.32	3126 +/- 37	-31.2	2.2
Stream	2015-11-24	-	-	-	-	-	66543	108.30±0.50	Modern	-28.2	13.4
Stream	2016-01-19	-	-	-	-	-	66546	106.42±0.49	Modern	-27.7	7.7
Stream	2016-03-30	-	-	-	-	-	67430	103.53±0.47	Modern	-28.7	14.4
Stream	2016-05-11	-	-	-	-	-	68222	111.44±0.51	Modern	-28.3	13.4
Stream	2016-06-14	-	-	-	-	-	68882	108.67±0.50	Modern	-28.4	10.8

Supplementary Figure 1: Timeseries of discharge through the a) upslope and b) riparian location, through the shallow layers (0 – 0.5 m below soil surface; light shades) and the deep layers (0.5 – 1 m below soil surface; dark shades) [1] and c) cumulative specific discharge (mm) for the stream and each of the four groundwater observation points.

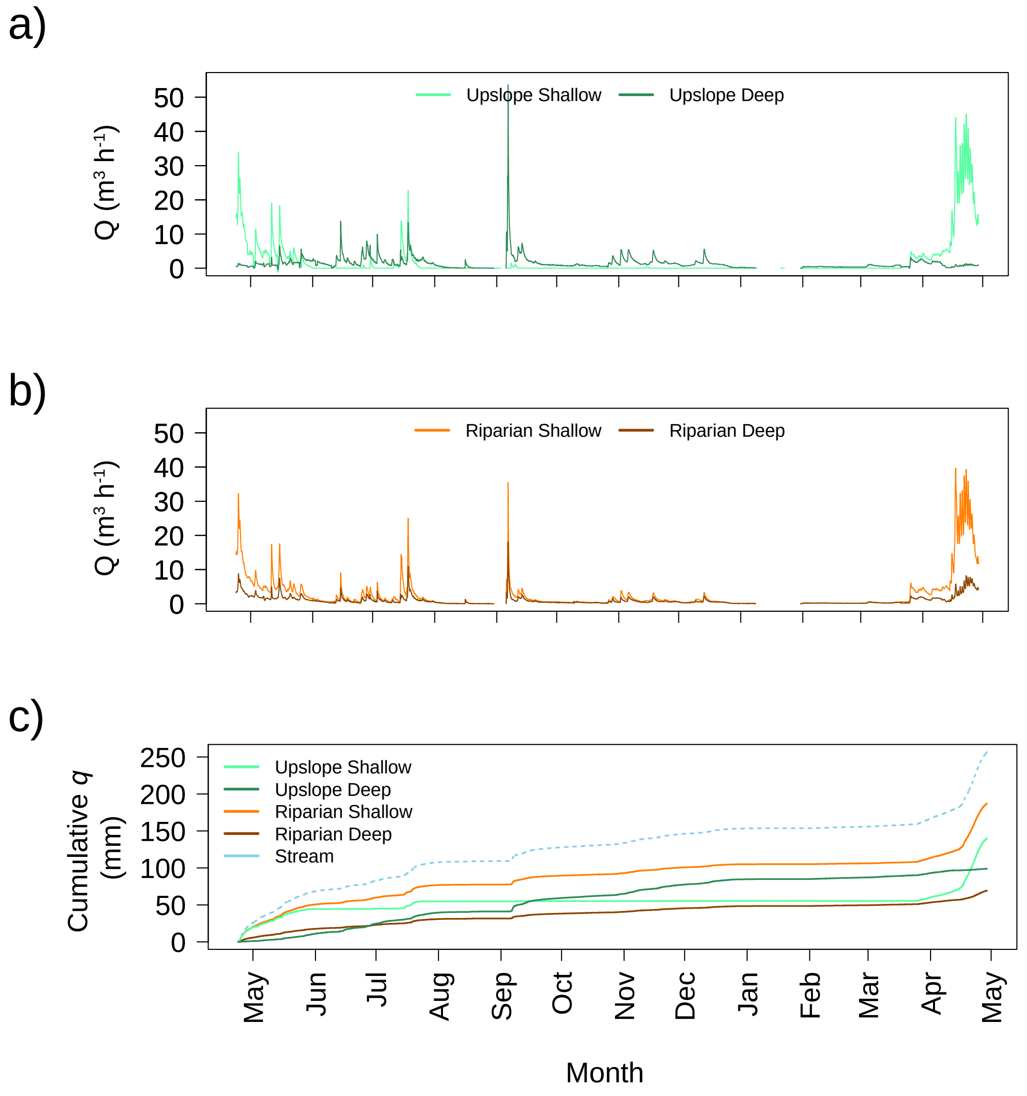

Supplementary Figure 2: Bi-plot showing the δ^{13}C values in (‰) against the ^{14}C content (% modern) of DOC (triangles) and CO_2 (circles), for this study and other published data, including the amazon river [2], soils and stream waters of northern peatland catchments [3, 4, 5] Data points included in this study are coloured according to their sampling locations; stream, riparian shallow (0-0.5m), riparian deep (0.5-1m), upslope shallow (0-0.5m), upslope deep (0.5-1m). End-members for organic matter (OM) derived from C3 and C4 plant material are identified in the grey rectangles. The atmospheric CO_2 end-members for the years 2015-2016 (this study) and 1991 to 2003 [2] are identified respectively by the red and pink rectangles. The carbonate rocks CO_2 end-member is also identified with an arrow in the bottom right corner of the figure, despite that such minerals are not present in our studied catchment.

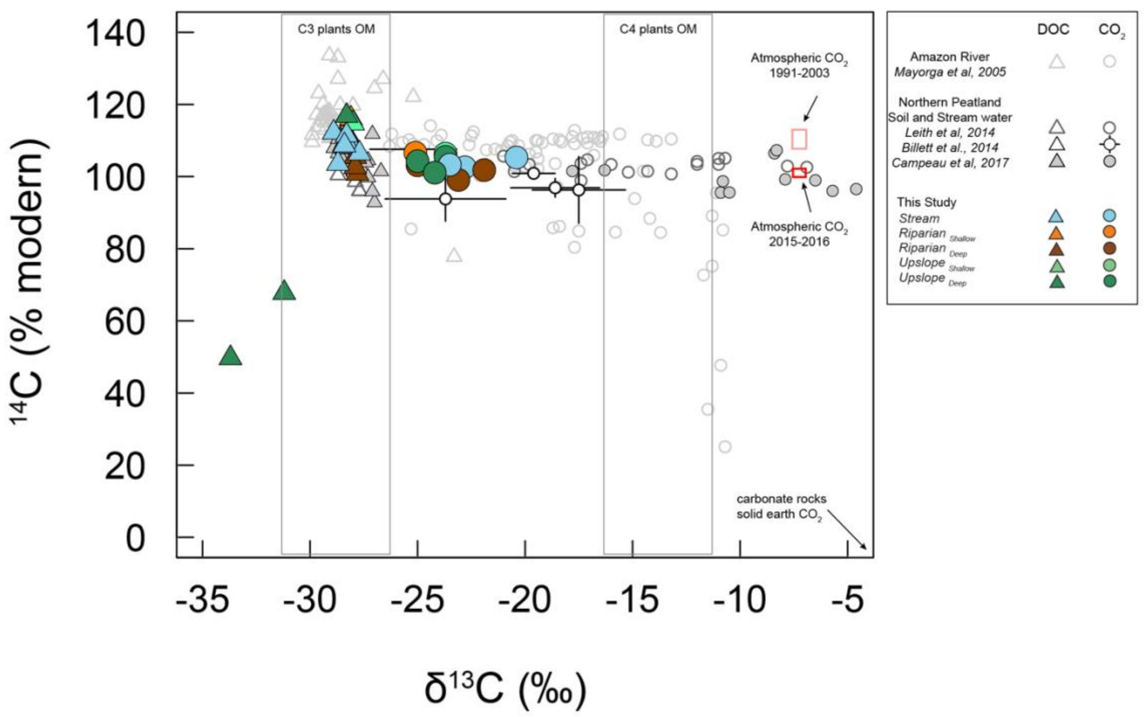

References

1. Amvrosiadi N, Seibert J, Grabs T, Bishop K. Water storage dynamics in a till hillslope: the foundation for modeling flows and turnover times. *Hydrological Processes* **31**, 4-14 (2017).

2. Mayorga E, *et al.* Young organic matter as a source of carbon dioxide outgassing from Amazonian rivers. *Nature* **436**, 538-541 (2005).

3. Leith FI, Garnett MH, Dinsmore KJ, Billett MF, Heal KV. Source and age of dissolved and gaseous carbon in a peatland–riparian–stream continuum: a dual isotope (^{14}C and $\delta^{13}C$) analysis. *Biogeochemistry* **119**, 415-433 (2014).

4. Campeau A, *et al.* Aquatic export of young dissolved and gaseous carbon from a pristine boreal fen: Implications for peat carbon stock stability. *Glob Chang Biol* **23**, 5523-5536 (2017).

5. Billett MF, Garnett MH, Dinsmore KJ. Should Aquatic CO_2 Evasion be Included in Contemporary Carbon Budgets for Peatland Ecosystems? *Ecosystems* **18**, 471-480 (2015).
